# CELLULAR RESILIENCE AND THE AGING HEART: UNDERSTANDING MITOCHONDRIAL INJURY MECHANISMS USING THE BABOON

**DOI:** 10.1093/geroni/igad104.3214

**Published:** 2023-12-21

**Authors:** Daniel Adekunbi, Elizabeth Reilly, Sandra Sanchez-Reilly, Cun Li, Peter Nathanielsz, Laura Cox, Adam Salmon

**Affiliations:** UT Health San Antonio, San Antonio, Texas, United States; Health Careers High School, San Antonio, Texas, United States; South Texas Veterans Health Care System, San Antonio, Texas, United States; Texas Biomedical Research Institute, San Antonio, Texas, United States; Texas Biomedical Research Institute, San Antonio, Texas, United States; Wake Forest University Health Sciences, Winston Salem, North Carolina, United States; UT Health San Antonio, San Antonio, Texas, United States

## Abstract

**Background:**

Aging-related cardiovascular disease is deadly worldwide. Cardiac aging in non-human primates (NHP), including baboons, is similar to humans, and intrauterine growth restriction animal models (IUGR) have shown to accelerate cardiac aging and foment cardiomyopathies. Objective: To measure and compare basal and metabolically stressed mitochondrial function among cardiac fibroblasts (CF) isolated from control vs. prematurely older (IUGR) baboons.

**Methods:**

CF were isolated from IUGR and control baboon hearts under standard conditions (25mM Glucose). Additional subgroups were exposed to Metabolic Stress (MS-exposed to 2-hours of low glucose, 1mM). Mitochondrial Oxygen Consumption Rate (OCR) and extracellular acidification rate (ECAR) were measured via Seahorse XFe96 instrument.

**Results:**

Donor Age: 13.27 – 17.52 years (elderly baboons), Male/Female data combined, n=4-5. OCR was higher in control relative to IUGR among all groups. ATP demand and non-mitochondrial respiration had similar values between control and IUGR. MS impacted CF bioenergetics similarly in control and IUGR. ECAR was higher in MS cells in both control/IUGR. OCR:ECAR ratio was higher in control vs IUGR while MS lowered OCR:ECAR ratio in control to levels comparable to IUGR.

**Conclusion:**

Higher OCR indicated increased metabolic demands of aging cells depending on glycolysis for survival. MS significantly impaired mitochondrial function across all cardiac cells regardless of aging. IUGR not only accelerated cardiac aging but impaired basal and metabolic response of CF. CF retain metabolic characteristics of their donor status and represent a cardiac aging model to explore mitochondrial injury mechanisms, understand cellular resilience and subsequently develop protective translational interventions.

